# Alleviation of glyphosate-induced toxicity by Horseradish tree (*Moringa oleifera*) Leaf extract and phytase in Nile Tilapia (*Oreochromis niloticus*) highlighting the antioxidant, anti-inflammatory, and anti-apoptotic activities

**DOI:** 10.1007/s11259-025-10672-5

**Published:** 2025-03-10

**Authors:** Esraa A. Elahwl, Doaa H. Assar, Ibrahim I. Al-Hawary, Abdallah S. Salah, Amany E. Ragab, Ahmed Elsheshtawy, Mona Assas, Haitham G. Abo-Al-Ela, Alamira Marzouk Fouad, Zizy I. Elbialy

**Affiliations:** 1 Fish processing and Biotechnology Department, Faculty of Aquatic and Fisheries Sciences, Kafrelsheikh University, Kafrelsheikh, 33516 Egypt; 2 Clinical Pathology Department, Faculty of Veterinary Medicine, Kafrelsheikh University, Kafrelsheikh, 33516 Egypt; 3 Department of Aquaculture, Faculty of Aquatic and Fisheries Sciences, Kafrelsheikh University, Kafrelsheikh, 33516 Egypt; 4 Institute of Aquaculture, Faculty of Natural Sciences, University of Stirling, Stirling, FK9 4LA UK; 5 Pharmacognosy Department, Faculty of Pharmacy, Tanta University, Tanta, 32527 Egypt; 6 Department of Aquaculture, Faculty of Fish Resources, Suez University, Suez, 43518 Egypt; 7 Aquatic Animal Medicine and Management, Faculty of Veterinary Medicine, Assiut University, Assiut, 71526 Egypt

**Keywords:** Horseradish, Glyphosate, Phytase, Nile tilapia

## Abstract

The danger posed by waterborne toxicity from herbicides endangers the aquatic ecosystem. Using dietary medicinal herbs is a useful approach to mitigate the effects of herbicide toxicity on aquatic animals. This study attempts to examine the consequences and potential mechanisms behind the dietary addition of horseradish tree (*Moringa oleifera*) leaf extract (MOLE) with the help of phytase addition to check the overall growth performance, biochemical changes, histological alteration, and gene expression in normal and after glyphosate challenge in Nile tilapia. A total number of 135 Nile tilapia fish (7.93 $\pm$ 0.03 g) were randomly assigned into three groups each in triplicate. The first group is the control group and fed basal diet; the second group supplied with MOLE (200 mg of extract/kg), and the third group was supplied with MOLE (200 mg /kg), and phytase (0.2g/ kg) for 8 weeks. After the feeding trial, each experimental group was divided into two subgroups to be unchallenged and challenged with glyphosate (30 mg/L of water). The results declared significant enhancements (*P* < 0.05) in Weight Gain Percent (WG%), Specific growth rate (SGR), and Protein efficiency ratio (PER) and reducing feed conversion ratio (FCR) with up-regulating hepatic *gh, igf1*,myogenine, intestinal ghrelin and *NPY* in fish groups fed MOLE and phytase compared with the control group. Moreover, improving the hepatic antioxidant capacity while down-regulating hepatic *igf1bp*, myostatin. Interstingly, MOLE and phytase lightened glyphosate-induced biochemical alterations, antioxidants, apoptosis, and inflammation-associated genes compared to the glyphosate-challenged group. Interestingly, UPLC-ESI-MS/MS analysis recognized 16 compounds encompasing two glucosinolates, three flavonoids, one phenolic and three alkaloids in addition to four fatty acids, a terpenoid, one phytate and an aromatic glycoside. These components might be accountable for the potential effects exerted by MOLE. Therefore, the current study suggests that dietary supplementation to MOLE and phytase can be used as substitute feed supplements in sustainable farming of Nile tilapia to defend against glyphosate challenges and enhance growth, antioxidant capacity, exerting anti-inflammatory and antiapoptotic effects under normal health conditions or post glyphosate challenge.

## Introduction

Aquaculture and agriculture are interconnected, with aquaculture often relying on agricultural drainage water due to limited water resources in some regions (Gewaily et al. 2021; Rossi et al. 2020). However, the extensive use of herbicides and insecticides in agriculture poses significant challenges to aquaculture, as waterborne chemicals adversely affect the health and productivity of aquatic species (Bojarski and Witeska 2020; Naiel et al. 2020; Dar et al. 2022). Glyphosate, a widely used herbicide with carcinogenic properties (Van Bruggen et al. 2018), induces oxidative stress, inflammation, apoptosis, and immunosuppression in fish (Ma et al. 2019; Mohapatra et al. 2020; Yalsuyi et al. 2021). Toxic chemicals from pesticides and herbicides damage vital tissues such as gills, skin, and intestines—key barriers in fish exposed to contaminated water (Banaee et al. 2020; Yang et al. 2020; Saha et al. 2021). Herbicide toxicity disrupts reactive oxygen species (ROS) balance, leading to lipid peroxidation, oxidative stress, and impairments in metabolic, physiological, and immunological functions (Yang et al. 2020; Sutili et al. 2020).

The production and growing application of glyphosate have resulted in its direct introduction into the environment. Several studies have indicated that glyphosate exhibits low bioaccumulation in the environment, as it is readily broken down by microbial activity and becomes inactivated through adsorption into the soil (Gandhi et al. 2021). Meanwhile, the use of glyphosate in aquatic environments can result in trace amounts being detected. However, due to its low vapor pressure (ranging from 1.84 × 10⁻⁷ mm Hg to 6.75 × 10⁻⁸ mm Hg at 298 K) and ionic nature, its presence from evaporation is minimal. Its occurrence in the air is primarily attributed to spray application and meteorological factors, which may impact nearby non-target plants as mentioned by Gandhi et al. (2021).

Aquaculturists have created cost-effective and highly nutritious fish diets that incorporate immunostimulants to maintain optimum fish health throughout the grow-out phases (Abidin et al. 2021). Additionally, the utilisation of plant-based components has gained attention of most fish nutritionists due to their affordability and local availability (Napier et al. 2020) in the same time it is well-known that aquaculture is one of the ideal industries that could benefit from the use of products and extracts obtained from different sopurces like citrus wastes (Kesbiç et al. 2022).

Currently, there is a focus on incorporating functional additives (eco-friendly) in aquatic feed to improve the physiological, metabolic, and immunological responses of aquatic animals (Elbialy et al. 2020, 2021; Elumalai et al. 2021). In this context, medicinal herbs have attracted significant interest due to their abundant bioactive compounds and their high functionality as well (Brum et al. 2018; Cardoso et al. 2021). The substantial importance of medicinal herbs focus on their pharmacological potential as natural antioxidative and anti-inflammatory agents indicates their potential use in mitigating the effects of insecticides, herbicides, and pesticides on aquatic animals (Mokhbatly et al. 2020; Sinha et al. 2021; Yousef et al. 2021).

Horseradish tree [*Moringa oleifera* (MO)] well-known for its high nutritional value, offering source of essential fibers, vitamins, minerals, proteins, and lipids (Mahfuz and Piao 2019). It exhibits various beneficial properties, including antidiabetic, anti-inflammatory, anticancer, antioxidant, antibacterial, and antifungal effects (Kamble et al. 2015; Coz-Bolaños et al. 2018; Ma et al. 2020; Mwamatope et al. 2020). The leaves of Moringa are rich in beneficial phytochemicals, such as tannins, terpenoids, alkaloids, isothiocyanates, sterols, flavonoids, saponins, glucosinolates, anthraquinones, and glycosides (González-Romero et al. 2022)**.**

Moringa leaf extract (MOL) was utilized in numerous researches to stimulate growth, enhance immune response, boost antioxidant activity, and protect against diseases in Nile tilapia in fry as well as different life stages (Elabd et al. 2019; Abd-El-Gawad et al. 2020; Chen et al. 2020), fingerlings (El-Kassas et al. 2020a, b; Emam et al. 2024), and adult fish (Hamed and El-Sayed 2019; El-Son et al. 2022). Additionally, some previous researches have shown the potential of Moringa leaves to reduce stress indices (Elabd et al. 2019; Hamed and El-Sayed 2019), as well as mitigate the sub-lethal toxicity of fipronil (Mahmoud et al. 2022) and sub-chronic sodium fluoride (Ahmed et al. 2020) in Nile tilapia.

Phytate is one of the anti-nutritional compounds present in Moringa leaves that is not possible to be removed by soaking or heating. High levels of phytic acid in different sources of plant protein negatively affect growth, nutrient retention, and mineral absorption (Gatlin et al. 2007). Studies have revealed that approximately 60–80% of phosphorus in plant by-products is in the form of phytate as chelated compound (Lei et al. 2013). Phosphorus in this form cannot be metabolized by mono-gastric and agastric fish, leading to nutrient runoff that contributes to aquatic pollution.

The addition of dietary phytase to feed is an effective technique to improve feed conversion ratio (FCR), enhance mineral absorption, promote thorough digestion, and increase phosphorus retention in the body, thereby reducing pollution in aquatic environments (Hussain et al. 2011; Liu et al. 2013; Hussain et al. 2015b).

In aquaculture, Nile Tilapia (*Oreochromis niloticus*) is emerging as a promising species for aquaculture, thanks to progressions in different techniques of hybridization and genetic engineering that enable its cultivation in various environments including fresh, brackish, and marine environments (Yue et al. 2016). The largest production of tilapia is recorded in China, followed by Indonesia, Egypt, Brazil, and Thailand (FAO 2022). As of 2020, Nile tilapia holds the third position among the major aquaculture species, with a production of 4.4 million tonnes (FAO 2022).

Despite studies on the effects of *M. oleifera* leaf extract (MOLE) on fish health status and growth performance (Abidin et al. 2022; Abdel-Latif et al. 2022; El-Kassas et al. 2022), there is limited knowledge on MOLE's ability to mitigate the toxicological effects of glyphosate on Nile tilapia (*O. niloticus*). The current research aims to explore the efficacy of MOLE and phytase on Nile tilapia growth performance and explore their impacts on glyphosate-induced oxidative stress, inflammation, apoptosis, and kidney and liver dysfunction.

## Material and methods

### Ethical statements

All experimental procedure were conducted in accordance with the Egyptian codes of ethics and was approved by the Committee of Animal Ethics at the Faculty of Aquatic and Fisheries Sciences, Kafrelsheikh University, Egypt (approval number: IAACUC-KSU-3–2022).

### Preparation of Moringa oleifera leaf extract

The leaves of M. oleifera were harvested from a private garden (April, 2021) and were authenticated by an expert in Botany. A voucher sample was kept (ID: Ziz300). To identify the plant more accurately, chemical characterization of the secondary metabolites was investigated using UPLC-ESI-MS/MS analysis.

The leaves were dried in the shade and were ground to a fine powder. The powdered plant (1 kg), was extracted by aqueous methanol (90%) for 3 days at room temperature with frequent shaking. The extract was then filtered to remove the powder. The extract was distilled off using a rotary evaporator to yield a brownish-green residue (yield 2%).

### UPLC-ESI–MS/MS

Ultra-performance liquid chromatography with electrospray ionization quadrupole-linear ion trap-tandem mass spectrometry analysis were implemented following a published procedure (Assar et al. 2023). It was performed on ESI-MS positive and negative ion acquisition mode using a XEVO TQD triple quadruple instrument. A multiple-reaction monitoring (MRM) mode was used for the quantitative determination of different phytochemicals. The crude extract of Moringa leaves was analyzed by UPLC. The collected samples were dissolved in methanol (HPLC-grade), filtered using a 0.2 μm-diameter membrane disc filter, the obtained solution concentration was in the range of 0.3mg/mL.

Waters mass spectrometer (Waters Corporation, Milford, USA) was used in the current study. The reverse-phase C18 column utilized was ACQUITY UPLC BEH (1.7 μm particle size, 1.7 μm–2.1 × 50 mm; 50 mm × 1.2 mm inner diameter) and 0.2 m/mL flow rate. A gradient elution program of solvent A (acidified water with 0.1% formic acid); solvent B (acidified methanol with 0.1% formic acid), was applied for the analysis. The elution conditions applied were: 0–2 min, isocratic elution (90% A); 2–5 min, linear gradient (90 to 70% A); 5–15 min, linear gradient (70% to 30% A); 15–22 min, linear gradient (30% to 10% A); and 22–25 min, isocratic elution (10% A); finally, column was washed and reconditioned. Electrospray ionization (ESI) was applied in both positive and negative ion modes. The parameters used for analysis were: source temperature of 150°C; cone voltage of 30eV; capillary voltage of 3 kV; desolvation temperature of 440°C; cone gas flow of 50 L/h; and desolvation gas flow of 900 L/hr.

Chemical components were identified using their ESI–MS2 spectra and fragmentation profiles. The raw data were analyzed by software MassLynx 4.1 and characterized by comparing both mass spectra and retention time (Rt) with the published data.

### Experimental design

A total number of 135 healthy mono-sex males of Nile tilapia fish (*Oreochromis niloticus*) 7.93 $\pm$ 0.026g (initial mean weight $\pm$ standard deviation) was reared in a private local fish farm at Kafrelsheikh governorate, Egypt. Collected fish were transported in oxygenated polyethylene bags to the biotechnology lab, Department of Fish Processing and Biotechnology, Kafrelsheikh University, Egypt and acclimated for a period of two weeks and fed normal fish diet by percentage of 5% of the body weight (Table 1) twice daily (09:00 AM and 2:00 PM) for two months. Aerators (stones) were added to make water saturation with oxygen and mechanical filters were used.

**Table 1:** Ingredients and proximate composition (g/kg, as-fed) of the control group, *Moringa oleifera* leaf extract and *Moringa oleifera* leaf extract with phytase enzyme

Ingredients (g/kg)	Basal	Moringa extract	Moringa + Phytase
Fish meal (65%)	10	10	10
Soybean meal (45%)	40.8	40.8	40.8
Corn gluten meal	6	6	6
Yellow corn	19.5	19.5	19.5
Wheat flour	18.5	18.5	18.5
Soybean oil	3	3	3
Vitamin mixture^*^	0.8	0.8	0.8
Mineral mixture^**^	0.5	0.5	0.5
DiCaP	0.6	0.6	0.6
Choline chloride	0.2	0.2	0.2
Stay C^***^	0.1	0.1	0.1
Moringa extract	0	0.2	0.2
Phytase enzyme	0	0	0.2
Composition (%)			
Crude protein	31.85	31.67	31.80
DE (Kcal/Kg)	3005.16	3004.84	3004.52
Crude lipid	5.44	5.56	5.72
Ash	4.923	4.925	4.927
Crude fiber	3.827	3.830	3.832
Ca	0.784	0.784	0.784
P	0.810	0.810	0.810

^*^Vitamin (g/kg premix): Thiamin HCl, 0.44; Riboflavin, 0.63; Pyridoxine HCl, 0.91; DL pantothenic acid, 1.72; Nicotinic acid, 4.58; Biotin, 0.21; Folic acid, 0.55; Inositol, 21.05; Menadione sodium bisulfite, 0.89; Vitamin A acetate, 0.68; Vitamin D3, 0.12; dL-alpha-tocoperol acetate, 12.63; Alpha-cellulose, 955.59

^**^Trace mineral (g/100 g premix): Cobalt chloride, 0.004; Cupric sulfate pentahydrate, 0.25; Furrous sulfate, 4.000; Magnesium sulfate anhydrous, 13.862; Manganous sulfate monohydrate, 0.650; Potassium iodide, 0.067; Sodium selenite, 0.010; Zinc sulfate hepahydrate, 13.193; Alpha-cellulose, 67.964

^***^Stay C, (L-ascorbyl-2-polyphosphate 35%)

^a^Moringa leaf extract 0.2 g/kg (200mg/kg)

^b^Phytase enzyme 0.2 g/kg (200mg/kg)

Water quality parameters were monitored throughout the study period using calibrated portable instruments. The parameters of water quality including pH, temperature, and dissolved oxygen were monitored daily and recorded as 26.23 ± 3°C, 5.8 ± 0.7 mg/L, and 7.60 ± 0.2, respectively while total ammonia was checked once per week. Dissolved oxygen (DO) was measured using a HI-9146-04 HANNA® galvanic DO meter (Hanna Instruments Ltd, Leighton Buzzard, UK), which employs an electrochemical method with automatic temperature compensation. Total ammonia nitrogen (TAN) was quantified using a HI-97715 photometer® (Hanna Instruments Ltd, Leighton Buzzard, UK), which utilizes the standardized Nessler method. pH and temperature were measured using an MW105 portable meter® (Milwaukee Instruments, Hungary), which employs the glass-electrode potentiometric method for pH determination.

Fish were divided into three groups equally distributed in 9 rectangular transparent glass tanks (70*60*40 cm), 15 fish per tank triplicates for each of the three groups. The duration of the feeding trial was **8 weeks.**

### The formulated diet

The basal diet was prepared according to NRC, formulated of 31.8% CP, all ingredients were ground into fine powder to compose three groups of experimental diet (control group with normal fish diet, MOLE group at 200mg of extract/kg of dry feed and MOLE (methanolic extract) at 200mg of extract/kg of dry feed with Phytase enzyme at 0.2g/ kg) (Table 1).

To form pellets, a machine of meat grinder was used to form pellets (2.33mm), and then the pellets were left to dry in the shade. The final diet was preserved in the refrigerator to avoid rancidity.

### Glyphosate herbicide challenge

The challenge was performed after the end of the feeding trial (8 weeks), each group of the three experimental groups was divided into non-challenged and challenged groups and named as follows: control group (Control group non-challenged); control positive group (control challenged group); MOLE (*Moringa oleifera* leaf methanolic extract non-challenged); MOLE challenged (*Moringa oleifera* leaf methanolic extract challenged with glyphosate herbicide); MOLE + phy (*Moringa oleifera* leaf methanolic extract with phytase enzyme non-challenged); MOLE + phy challenged (*Moringa oleifera* leaf methanolic extract with phytase enzyme challenged with glyphosate herbicide). The challenge was performed by using commercial formulation Roundup® glyphosate herbicide (30 mg/l of water) by injection of herbicide in water for three days, when water was exchanged the herbicide was added to water again for each treatment and after three days all samples were collected from all treatments challenged and non-challenged to perform different analysis, and the exact dose of glyphosate (Roundup 48%, Agrochem, Alwatneia Co., Alex., Egypt) used in our study was kept at the necessary concentration of 0.6 mg/L in each aquarium. Concurrently, the control group's water was refreshed with dechlorinated tap water continuously. The lethal dose (LC50; 12 mg/L) of glyphosate was previously determined by Abdelmagid et al. (2021), where fish were exposed to 1/20 of the LC50 (0.6 mg/L) as per Abdelmagid et al. (2021).

### Growth parameters

Counting the weight of fish at the onset of the experiment to record the initial weight and every two weeks till the termination of the experiment to record fish final weight to determine the growth parameters: Weight gain, percent weight gain, total feed intake, specific growth rate, feed conversion ratio, survival rate, and protein efficiency ratio according to Assar et al. (2023).

The growth parameters were determined as follows:

- Body weight gain (BWG, g) = FBW–IBW
- Specific growth rate (SGR) (% body weight gain / day) = ((Ln FW − Ln IW) /t) × 100
- Feed conversion rate (FCR) = Feed intake (g) / Weight gain (g)
- Protein efficiency ratio (PER) = Live weight gain (g)/ Dry protein intake (g)

At the completion of the experimental period (8 weeks), 9 fish/group were randomly selected, the growth performance, efficiency feed utilization, biochemical, pathological, and molecular analysis were calculated, followed by glyphosate challenge which was conducted for an additional 3 days.

### Blood sampling,and tissue collection

After eight weeks, 9 fish/experimental group (from both glyphosate-challenged and nonchallenged subgroups) were randomly selected and anesthetized by 150 mg/L MS222 (Argent Laboratories, Redmond, Washington). Using a disposable plastic syringe, blood samples were collected by caudal puncture in a plain Eppendorf tube without anticoagulant, after coagulation samples were centrifuged at 3000 rpm/15 min at 4 ◦C, divided into multiple aliquots, and stored at − 20◦C for serum biochemical parameters assessment. Biochemical measurements were performed using a spectrophotometer and commercial kits (BioDiagnostic, Cairo, Egypt). Immediately after blood collection from each fish tissue samples from the gills, liver, and intestine were rapidly excised, and collected from each fish were kept in 10% neutral buffered formalin for further histological examination or shocked in liquid nitrogen and then stored at −80°C for further RNA extraction and gene expression measuring.

### Serum biochemical analysis

Serum samples were analyzed for liver injury biomarkers as ALT (alanine transaminase) and AST (aspartate aminotransferase) (Reitman and Frankel 1957), TC (total cholesterol|) (Allain et al. 1974), TG (triglycerides) (Fredrickson et al. 1967), total protein, albumin (Lowry et al. 1951; Doumas et al. 1971) respectively, BUN (blood urea nitrogen) (Patton and Crouch 1977) and creatinine (Henry 1974) were also measured. VLDL-C (Very-low-density lipoprotein) concentration was determined using the standard equation (Friedwald et al. 1972). Serum globulin concentrations (Glob) were estimated by subtracting the albumin concentration from the total protein amount, considering the albumin to globulin ratio (A/G) as described by Kaneko (1989).

### Histopathological examination

Fish samples were collected from each experimental group (9/group) for histopathological analysis and the target organs were gills, liver, and intestine. The target organs were kept in 10% neutral buffered formalin at room temperature for further analysis (Thermo Fisher, Kalamazoo, MI) for 48 h. On after one day day, the organs were washed several times with water and dehydrated in 75% ethyl alcohol. Gills, liver and intestine samples were dissected and observed along with standard histological techniques. Longitudinal sections of 5 μm increments were prepared from all tissues, fixed on glass slides, and were stained with hematoxylin and eosin (H&E) for further investigation using light microscope (Bancroft and Gamble 2008).

### Molecular analysis

Tissue samples were collected from each experimental group (9/group) were obtained to perform molecular analysis to study gene expression in the liver and intestine; tissue samples were collected in separate sterile Eppendorf tubes then instantly shocked in liquid nitrogen to perform RNA extraction.

Fifty mg liver and intestine samples in triplicates were used for total RNA extraction and was performed by using GENEzol™ Reagent (Geneaid,UK) according to manufacturer’s instructions. BioDrop spectrophotometer (Biochrom Ltd, UK.) was used to assess concentration and purity of RNA and the integrity of the extracted RNA was analyzed by using agarose gel electrphoresis. Fixed amount of RNA samples 5 μg were reverse transcriped by using cDNA master mix to get cDNA samples was performed by using TOPscript™ RT DryMIX kit (enzynomics, Korea).

Real-time PCR assay was accomplished by using TOPreal™ SYBR Green qPCR PreMIX (enzynomics, korea) for specific genes of growth, antioxidant, inflammatory and apoptosis as Growth hormone gene (GH), Insulin like growth factor (IGF), Insulin like growth factor binding protein (IGFBP), Myostatin (MYos), Myogenin (MYoG), Superoxide dismutase (SOD), Cyclooxygenase2 (COX2), Caspase3 (Cas3), Neuropeptide Y (NPYa) and Ghrelin (Ghr), by using gene specific primer sequences in liver and intestine for these genes (Table 2). 10µL of master mix (0.6 µL of forward primer, 0.6 µL of reverse primer, 1 µL cDNA template, and 7.8 nuclease-free water) and 10 µL of SYPER to make final volume 20 µL. Steps were performed by using Rotor Gene-Q (QIAGEN, Germany) cycler with the following: Activation step for 15 min at 95°C, Denaturation for 10 s at 95°C, annealing for 15 s at a specific temperature depending on type of primer, and the extension step at 25 s for 72°C.

**Table 2:** Primers’ sequences used for Q-rtPCR

	Gene	Primer sequence (5'−3')	Accession number	Reference
Internal reference genes				
1	*ef1a*	For: GCACGCTCTGCTGGCCTTT Rev: AGCCAGACGGACAGATGCC	AB075952	Yang et al. (2013)
2	*gapdh*	For: GATAATGGCAAACTTGTCGTCG Rev: ACATTGGAGCATCGGGTGAG	JN381952	Yang et al. (2013)
Growth related genes				
3	*gh*	For: GTTGTGTGTTTGGGCGTCTC Rev: CAGGTGCGTGACTCTGTTGA	HM565014.1	Abo-Raya et al. (2021)
4	*igf-1*	For: TCCTGTAGCCACACCCTCTC Rev: ACAGCTTTGGAAGCAGCACT	NM_001279503.1	Costa et al. (2016)
5	*igfbp1a*	For: TCCTAGACCTGGTGAAGCCA Rev: CGAGGTCG ACAGTGC AGATT	XM_003438121.3	Assar et al. (2024)
6	*myost*	For: GCATCTGTCTCAGATCGTGCT Rev: TGCCATCATTACAATTGTCTCCG	KT987208.1	Elkatatny et al. (2016)
7	*myog*	For: GCAGCCACACTGAGGGAGAA Rev: AAGCATCGAAGGCCTCGTT	GU246717.1	Nebo et al. (2013)
Anti-oxidant related gene				
8	*sod*	For: CATGCCTTCGGAGACAACAC Rev: ACCTTCTCGTGGATCACCAT	AY491056.1	Han et al. (2016)
Inflammatory related gene				
9	*cox2*	For: AGCAGCCAGAAGGAAGGCGG Rev:GACTGAGTTGCAGTTCTCTTAGTGTGC	-	Chuang and Pan (2011)
Apoptosis related gene				
10	*cas3*	For: GGCTCTTCGTCTGCTTCTGT Rev: GGGAAATCGAGGCGGTATCT	GQ421464.1	Standen et al. (2016)
Control of feeding related gene				
11	*npya*	For: ACAAGACAGAGGTATGGGAAGA Rev: GGCAGCATCACCACATTG	XM_003448854.1	Yan et al. (2017)
Appetite stimulant related gene				
12	*ghr*	For: GCAGAAGACTTGGCGGACTACAT Rev: ATAAACCAGAAAGAAGGGACAACC	AB104859.1	Dong et al. (2016)

*EF1a* Elongation factor 1 alpha (internal reference gene), *GAPDH* Glyceraldehyde-3-phosphate dehydrogenase (internal reference gene), *GH* Growth hormone, *IGF1* Insulin-like growth factor, *IGFBP1a* Insulin-like growth factor binding protein 1a, *MYoS* Myostatin, *MYoG* Myogenin, *SOD* Super oxide dismutase, *COX-2* Cyclooxygenase 2, *Cas3* Caspase3, *NPYa* Neuropeptide Y, *Ghr* Ghrelin

The resulting curves were analyzed to assess the efficiency of amplification at melting temperature by showing one peak for all genes. Relative expression for all samples was evaluated by the method of 2–ΔΔCT (Livak and Schmittgen 2001). The last step was normalization of fold change for all genes against the housekeeping genes (EF1A and GAPDH genes).

### Statistical analysis

All data were expressed as mean ± standard error of mean (M ± SEM). Preliminary, data normality was assessed using Shapiro–Wilk’s test, while variance homogeneity was tested by Levene’s test, both performed with significance level at *p* ≤ 0.05. Percentage data were subjected to Arcsine transformation for the analysis of variances. To investigate the differential effects of MOLE, MOLE with phytase enzyme growth performances was analyzed by one-way ANOVA, biochemical analyses, histomorphometric measurements, and relative gene expression were analyzed using Two-way ANOVA, pursued by Tukey’s multiple comparison test (*p* ≤ 0.05). All statistical analyses were performed using GraphPad Prism (version 9.5, GraphPad Software, San Diego, USA).

## Results

### Phytochemical characterization of Moringa olifera

**Fig. 1:**
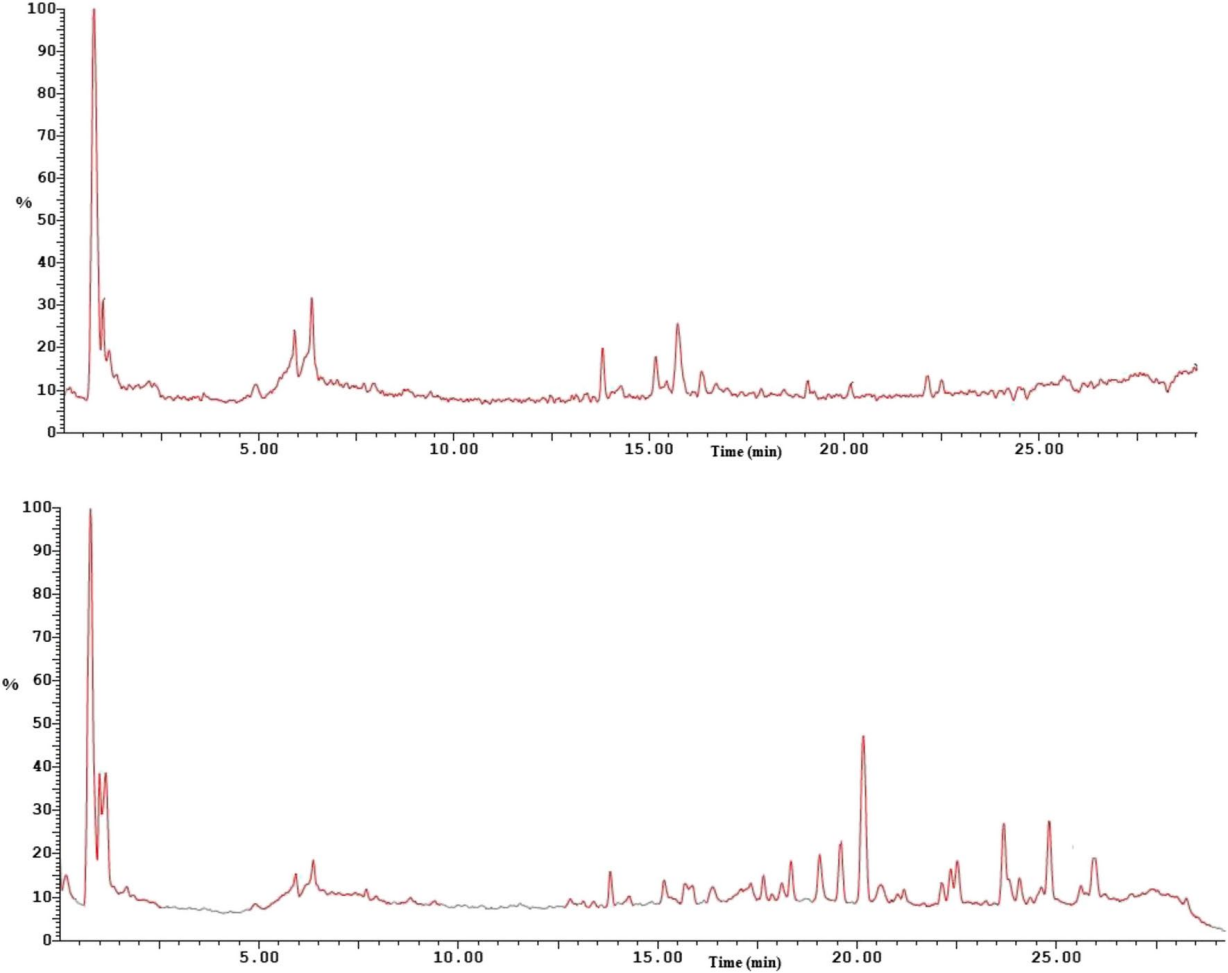
UPLC-ESI-MS base peak ion chromatograms of MOLE (negative ion mode: top, positive ion mode: bottom)

The UPLC-ESI-MS/MS examination in both positive and negative ion modes (Fig. 1) was utilized to help identifying the *Moringa* species in our study. This analysis identified 16 compounds (Table 3) based on their m/z and MS/MS fragmentation ions which are in accordance to the published data as follows. Of these compounds, two glucosinolates were identified namely glucomoringin and 4-(2’-*O*-acetoxy-α-L-rhamnopyranosyloxy) benzyl glucosinolate. Both compounds were identified previously in the leaves of *M. oleifera and M. stenopetala* (Maldini et al. 2014; Lin et al. 2019; Sibhat et al. 2023; Bennett et al. 2003, EFSA 2019). Chlorogenic acid, quercitin, isoquercitin and Kaempferol-3-*O*-rhamnoside were recognized in our study. These polyphenolic compounds were previously detected in the leaves of both *M. oleifera* (Lin et al. 2019, Zhu et al. 2020; Coppin et al. 2013; Bennett et al. 2003, Kumar et al. 2021)*.* Of these polyphenolic compounds, only chlorogenic acid was detected in both species *M*. *oleifera* and *M*. *stenopetala* (Bennett et al. 2003; Sibhat et al. 2023), while the flavonoids quercitrin, isoquercitrin and kaempferol-3-*O*-rhamnoside was only identified from the leaves of *M*. *oleifera*. Alkaloids marumoside B, tangutoride E, and niazirin were characterized in this research. These compounds were detected previously in the extract of the leaves of *M. oleifera* only (Lin et al. 2019; Sahakitpichan et al. 2011, EFSA 2019, Kumar et al. 2021). Consequently, the genus of *Moringa* in our study is *M. oleifera*.

**Table 3:** Phytochemical profiling of *M. oleifera leaves* by LC–ESI–MS/MS in negative and positive ion modes

NO	R_t_ min	[M-H]^−^ *m/z*	[M + H]^+^ *m*/*z*	MS^2^ Ions *m/z*	Identification	class
1	0.75		261	179	Inositol monophosphare	Phytate
2	1.00	570		97	Glucomoringin	Glucosinolate
3	1.12	504 [M + HCOO]^−^		427, 307, 279, 150	Marumoside B	Alkaloid
4	1.35	353		253, 190, 144, 125	Chlorogenic acid	Phenolic
5	2.38	612		259	4-(2’-*O*-Acetoxy-α-L-rhamnopyranosyloxy)benzyl glucosinolate	Glucosinolate
6	4.96	324		188, 147, 114, 88	Niazirin	Alkaloid
7	5.93		303	285, 212, 176,	Tangutoride E	Alkaloid
8	5.93	463		318, 300, 271, 178, 159	Isoquercetin	Flavonoid
9	6.36	447	449	300, 271	Quircitrin	Flavonoid
10	6.46	447 [M + HCOO]^−^		267, 163, 115	Kaempferol-3-*O*-rhamnoside	Flavonoid
11	9.41	329		293, 226, 212, 168, 137	Sanleng acid	Fatty acid
12	13.81	265		247, 211, 180, 169, 133	4-α,6-α-Dihydroxyeud-esman-8β−12-olide	Terpenoid
13	14.30	293		275, 247, 232, 152	(E,E)−9-Oxooctadeca-10,12-dienoic acid	Fatty acid
14	18.10		425	296, 253, 146, 73	Benzyl-O-β-D-xylopyranosyl-(1--−6)-β-D-glucopyranoside	Aromatic glycoside
15	19.05		277 [M + Na]^+^	237, 97, 88, 69	Palmitoleic acid	Fatty acid
16	22.35		284		Octadecanamide	Fatty acid derivative

### Growth performance

Concerning parameters for growth performance of Nile tilapia fed diets supplementd with MOLE and phytase are shown in Table 4. Fish growth traits ( FBW, WG%, SGR) were highest (*p* < 0.05) in the fish group fed the diet supplied with both MOLE and phytase compared to the control group. Regarding Feed utilization, the MOLE and phytase group showed a considerably (*p* < 0.05) lower FCR and higher PER matched to the control. Conversely, growth was markedly lowered (*p* < 0.05) in the fish group fed MOLE alone compared to the control. The survival rate and feed intake at the experiment did not show significant differences (*p* > 0.05) amongst the experimental diets.

**Table 4:** Growth and feed utilization parameters of Nile tilapia (*Oreochromis niloticus*) fed on normal fish diet, *Moringa oleifera* methanolic extract and *Moringa oleifera* methanolic extract with phytase enzyme for 8 weeks

Parameters	Control	Moringa	Moringa + phytase	*P* Value
IBW (g)	7.91 $\pm$ 0.01	7.91 $\pm$ 0.02	7.96 $\pm$ 0.01	0.7290
FBW (g)	24.42 $\pm$ 0.1^b^	20.33 $\pm$ 0.1^c^	27.04 $\pm$ 0.16^a^	0.0002
BWG %	208.73 $\pm 6.76$^b^	156.97 $\pm 3.96$^c^	240.0 $\pm$ 8.31^a^	0.0003
Total feed intake (g)	375.76 $\pm$ 2.11	375.76 $\pm$ 2.16	377.87 $\pm 2.14$	0.7290
SGR (% day-1)	1.86 $\pm$ 0.33^b^	1.56 $\pm$ 0.33^c^	2.06 $\pm$ 0.33^a^	0.0001
FCR	1.82 $\pm$ 0.02^ab^	2.4 $\pm$ 0.23^a^	1.57 $\pm$ 0.09^b^	0.0175
PER	1.76 $\pm$ 0.03^ab^	1.3 $\pm$ 0.13^b^	2.03 $\pm$ 0.12^a^	0.0094
Survival (%)	88.9 $\pm 2.2$	91.13 $\pm 4.43$	88.9 $\pm 2.2$	0.9328

Growth and feed utilization parameters of Nile tilapia (*Oreochromis niloticus*) fed on normal fish diet, *Moringa oleifera* methanolic extract and *Moringa oleifera* methanolic extract with phytase enzyme for 8 weeks

*IBW* Initial body weight, *FBW* final body weight, *BWG* body weight gain, *SGR* specific growth rate, *FCR* Feed conversion ratio, *PER* protein efficiency rate. Data are expressed as Mean ± SEM where *n* = 3 as triplicate tanks for BWG%, FCR, SGR, PER and *n* = 45 for IBW and FBW. Values with different superscripts within a row are significantly different (*p* < 0.05)

### Biochemical findings

The effects of the dietary MOLE and phytase on the serum biochemical parameters of the Nile tilapia are demonstrated in Table 5. The addition of MOLE and phytase in the diet significantly increased the concentration of total protein and globulin paralleled to the control groups, both before and after the challenge with glyphosate.

**Table 5:** Biochemical parameters of Nile tilapia (*Oreochromis niloticus*) reared for 8 weeks and fed on normal fish diet, *Moringa oleifera* methanolic extract and *Moringa oleifera* methanolic extract with phytase enzyme

Parameters	Control	MOLE	MOLE + phytase Enzyme	Control challenged	MOLE challenged	MOLE + phytase challenged	*p* value of two-way ANOVA		
							challenge	treatment	Interaction
Total protein (g/dL)	4.25$\pm$0.019^c^	4.62$\pm$0.02^b^	5.45$\pm$0.06^a^	4.155$\pm$0.01^c^	$4.566\pm$0.02^b^	5.37$\pm$0.03^a^	0.0077	< 0.0001	0.5975
Albumin (g/dL)	2.4$\pm$0.01^a^	2.42$\pm$0.02^a^	2.41$\pm$0.02^a^	2.30$\pm 0.02$^b^	2.3$9\pm$0.01^a^	$2.4\pm$0.04^a^	0.0080	< 0.0001	0.7972
Globulins (g/dL)	1.85$\pm$0.01^c^	$2.20\pm$0.02^b^	$3.04\pm$0.06^a^	1.86$\pm 0.01$^c^	2.18$\pm$0.02^b^	$2.97\pm$0.02^a^	0.0495	< 0.0001	0.5833
A/G	$1.29\pm$0.01^a^	1.1$\pm$0.02^a^	0.79$\pm$0.06^b^	$1.24\pm$0.01^a^	1.096$\pm$0.01^a^	0.81$\pm$0.02^b^	0.0762	< 0.0001	0.3429
VLDL (mg/dL)	23.75$\pm$0.04^c^	22.69$\pm$0.19^d^	21.86$\pm$0.01^e^	25.27$\pm 0.07$^a^	$24.88\pm$1.83^ab^	24.57$\pm 0.15$^b^	< 0.0001	< 0.0001	0.0037
Cholesterol (mg/dL)	115.93$\pm$0.47^b^	105.04$\pm$1.13^d^	103.69$\pm 0.53$^d^	125.30$\pm$0.52^a^	115.87$\pm$1.11^b^	110.96$\pm$1.40^c^	< 0.0001	< 0.0001	0.2821
Triglycerides (mg/dL)	118$.77\pm$0.78^c^	113.44$\pm$0.98^d^	109.3$0\pm 0.56$^e^	126.376$\pm$0.38^a^	124.4$\pm$0.92^ab^	122.86$\pm$0.77^b^	< 0.0001	< 0.0001	0.0037
Creatinine (mg/dL)	0.93$\pm$0.02^c^	0.87$\pm$0.01^d^	0.78$\pm$0.01^e^	1.28$\pm$0.04^a^	1.13$\pm$0.04^a^	1.03$\pm$0.01^b^	< 0.0001	< 0.0001	0.0003
Blood Urea nitrogen (mg/dL)	16.81$\pm$0.03^c^	15.14$\pm$0.011^d^	14.63$\pm$0.02^e^	21.51$\pm$0.04^a^	19.20$\pm 0.02$^b^	18.90$\pm$0.05^b^	< 0.0001	< 0.0001	< 0.0001
Ast (U/L)	34.20$\pm$0.24^d^	35.36$\pm$0.43^d^	33.5.$\pm$0.47^d^	55.16$\pm$0.25^a^	50.6$\pm$0.47^b^	42.73$\pm$0.48^c^	< 0.0001	< 0.0001	0.1165
Alt (U/L)	28.03$\pm$0.08^c^	28.19$\pm$0.26^c^	$26.63\pm$0.26^d^	44.1$\pm$0.12^a^	43.27$\pm$0.39^a^	$35.98\pm$0.31^b^	< 0.0001	< 0.0001	< 0.0001

Biochemical parameters of Nile tilapia (*Oreochromis niloticus*) reared for 8 weeks and fed on normal fish diet, moringa oleifera methanolic extract and *Moringa oleifera* methanolic extract with phytase enzyme for 8 weeks for total protein, albumin, globulin, albumin/globulin (A/G) ratio, very low-density lipoprotein (VLDL), cholesterol, triglycerides, Urea, creatinine, aspartate aminotransferase (AST) and alanine transaminase (ALT). Data are expressed as Mean ± SEM where *n* = 5. small letters indicate significant differences (two-way ANOVA). ** These parameters were measured in serum. Small letters indicate significant differences (two-way ANOVA). Values with different superscripts within a row are significantly different (*p* < 0.05)

Regarding the serum lipid profile as shown in Table 5, TC, TG, and VLDL-c levels showed significant reduction in the fish group fed MOLE either alone or with phytase compared to the control groups either with or without challenge, while they were markedly elevated in the glyphosate challenged group matched to the control group.

Concerning serum kidney injury biomarkers, BUN and creatinine concentrations showed significant reduction in the fish group fed the MOLE and phytase compared to the control groups either with or without glyphosate challenge, while they were significantly elevated in the glyphosate challenged group compared to the control group.

Furthermore, serum enzyme activity (ALT and AST) were analogous in MOLE, MOLE and phytase in relation to the control group. While, after the glyphosate challenge, the fish fed the MOLE and phytase exhibited lower activities paralleled to the control challenged group (*p* < 0.05).

### Histological findings

**Fig. 2:**
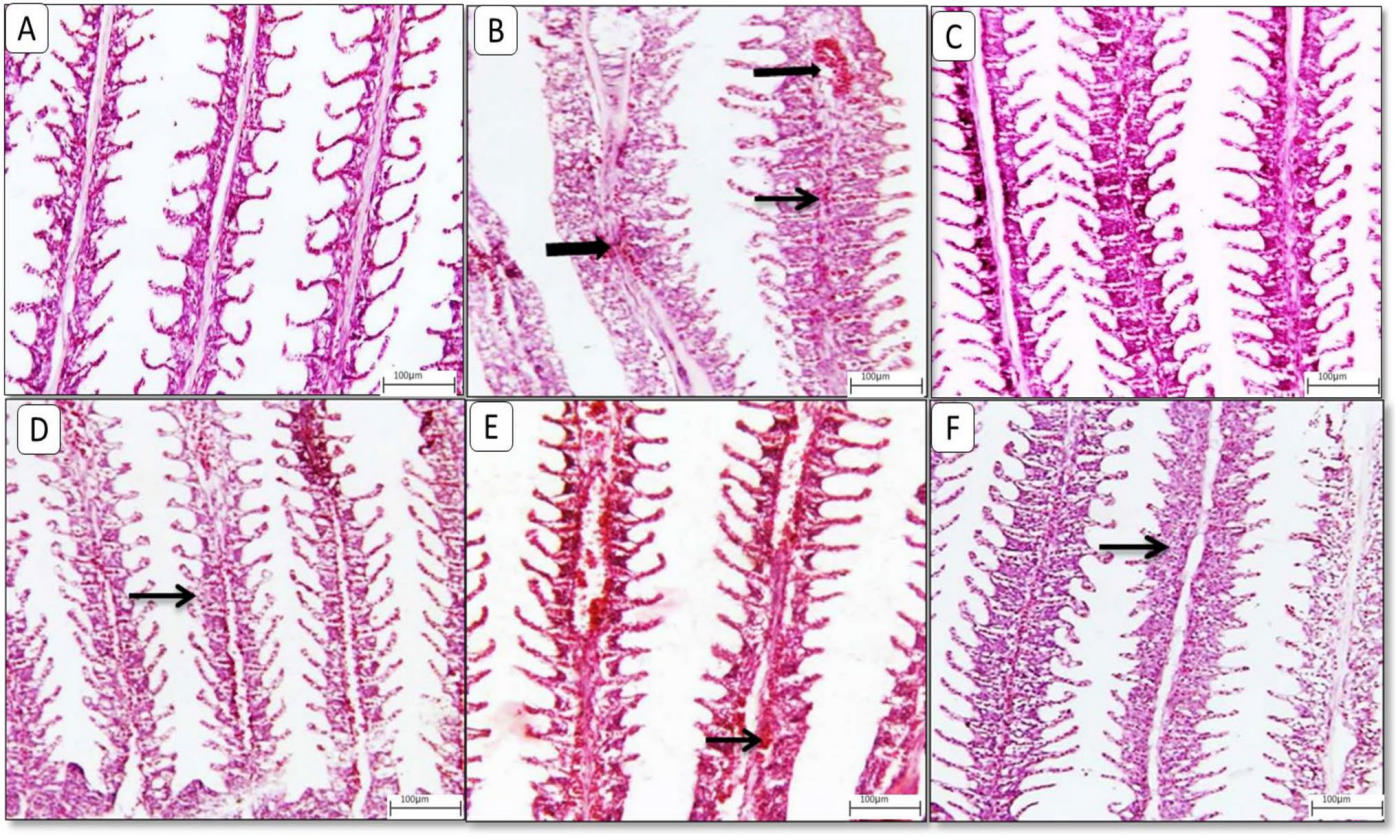
Representative photomicrograph of **gills** from different treatment groups. **A**) **Control** gills showing normal histological appearance of primary and secondary lamellae. **B**) **Glyphosate Challenged group** showing diffuse lamellar fusion (thin arrow) with lamellar congestion and hemorrhage (thick arrow). **C**) **MOLE** showing normal histological appearance. **D**) **MOLE challenged with glyphosate** group showing focal lamellar thickening (thin arrow) with interlamellar congestion (thick arrow). **E**) **MOLE and phytase** showing lamellar congestion with focal lamellar fusion (thin arrow). **F**) **MOLE and phytase challenged with glyphosate** showing moderate to severe lamellar thickening (thin arrow) with lamellar fusion besides lamellar lifting. Image magnification = 100x

**Fig. 3:**
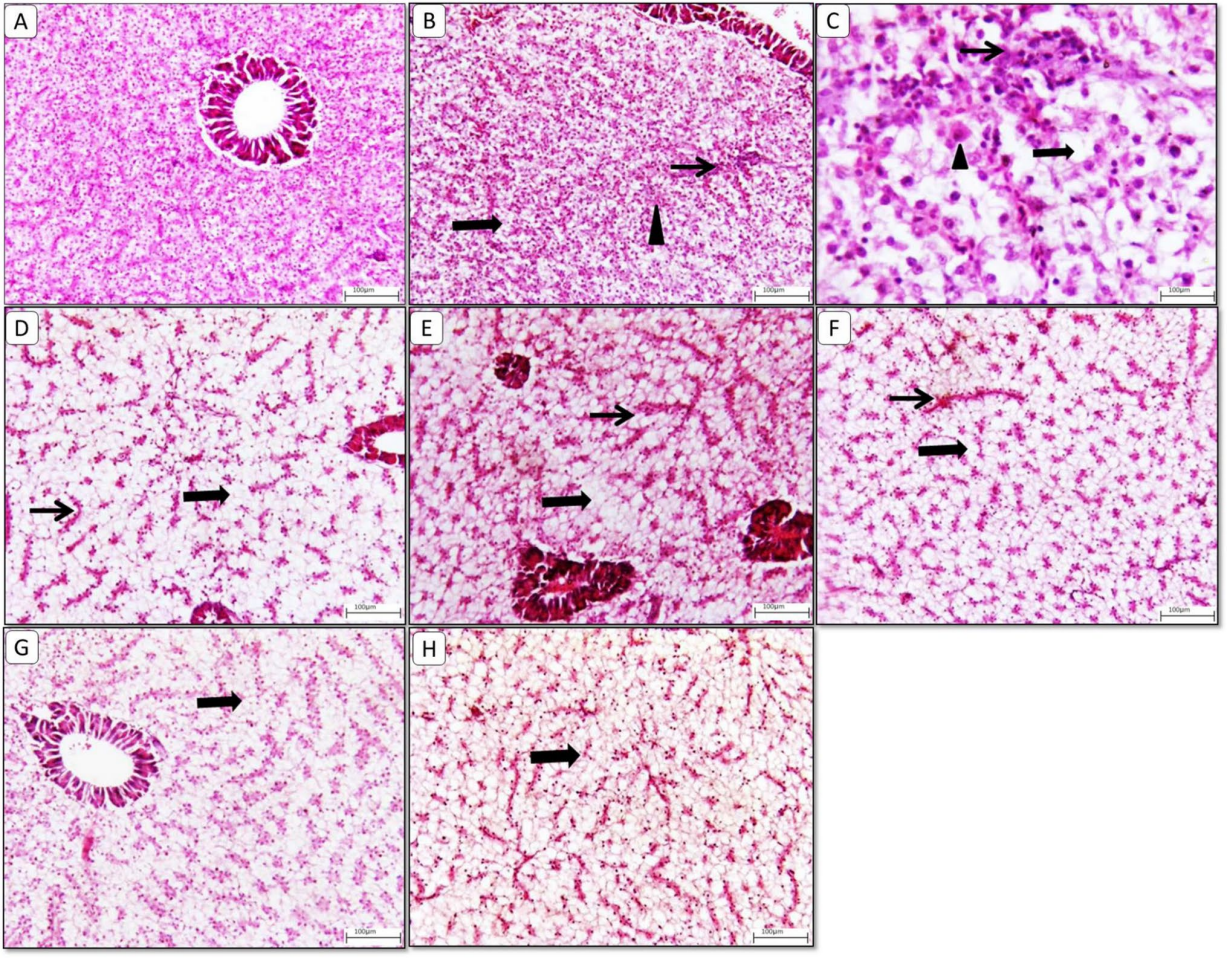
Representative photomicrograph of **liver** from different treatment groups. **A**) **Contro**l liver showing normal histological appearance of hepatic parenchyma and hepatopancrease. **B**) **Glyphosate Challenged group** showing diffuse swollen hepatocytes (thick arrow) with multifocal coalescing hepatocyte necrosis (arrowhead) and inflammatory aggregates (thin arrow). **C**) **higher magnification** showing necrotic hepatocytes (arrowhead) surrounded with inflammatory aggregates (thin arrow) beside diffuse hepatic vacuolation (thick arrow) and sinusoidal congestion. **D**) **Glyphosate Challenged group** showing diffuse extensive hepatic vacuolation (thick arrow) and minimal sinusoidal congestion (thin arrow). **E**) **MOLE** showing moderate hepatic vacuolation (thick arrow) with sinusidal congestion (thin arrow). **F**) **MOLE challenged with glyphosate** showing diffuse hepatic vacuolation (thick arrow). **G**) **MOLE and phytase** showing moderate hepatic vacuolation (thick arrow). **H**) **MOLE and phytase challenged with glyphosate** showing diffuse severe hepatic vacuolation (thick arrow). Image magnification = 100x

**Fig. 4:**
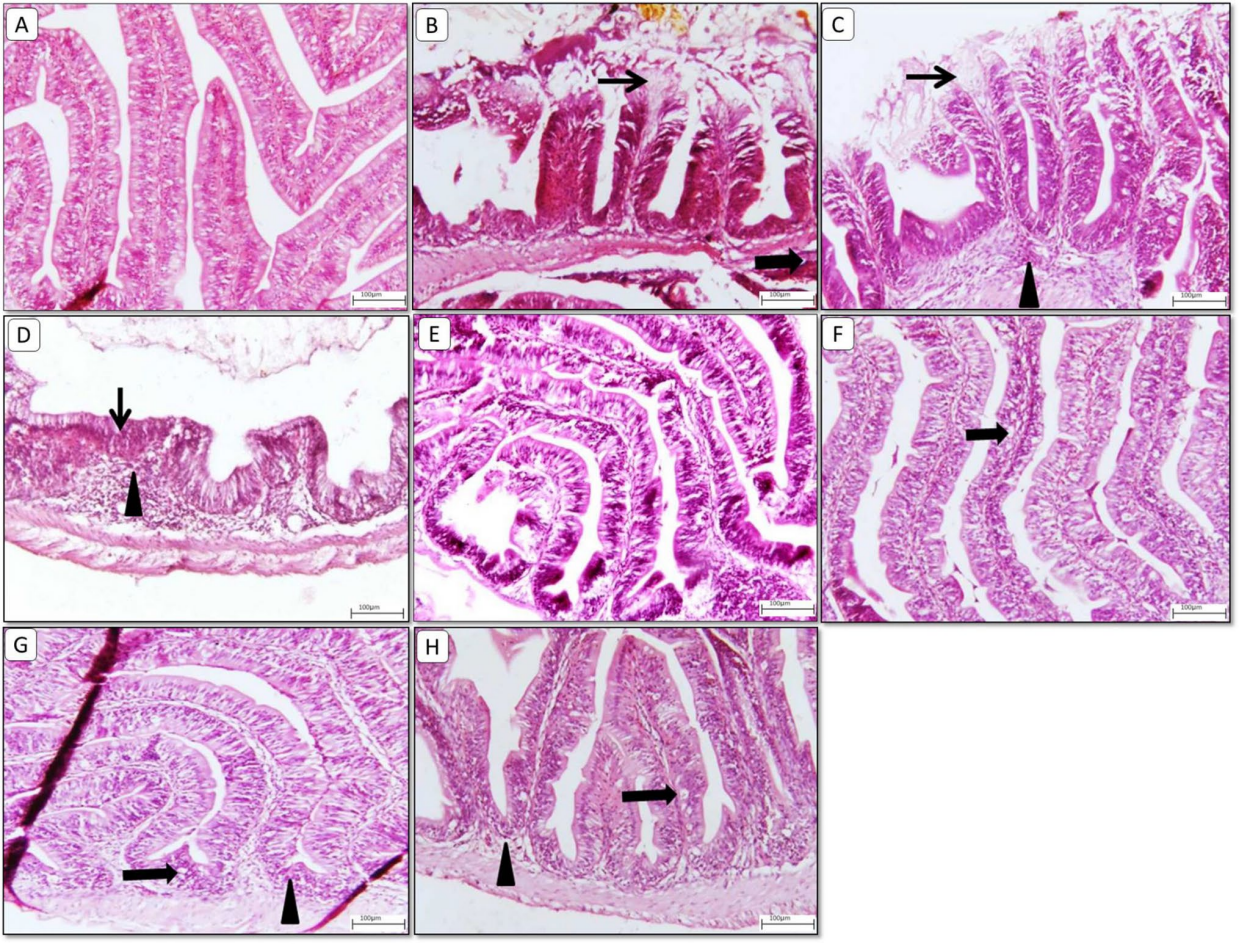
Representative photomicrograph of **intestinal** section from different treatment groups. **A**) **Control** group showing normal histological appearance of intestinal villi. **B**, **C**) **Glyphosate Challenged group** showing extensive apical desquamation (thin arrows) with mild lamina proprial inflammatory aggregates (arrowhead). **D**) **Glyphosate-Challenged group** showing diffuse shortened, stunted fused villi (thin arrow) with marked lamina proprial cellular infilterates (arrowhead) and submucosal edema. **E**) **MOLE** showing normal histological appearance. **F**) **MOLE challenged with glyphosate** showing mild intestinal vacuolation (thick arrow). **G**) **MOLE and phytase** showing few intestianl vacuolation (thick arrow) with few invaded inflammatory cells (arrowhead). **H**) **MOLE and phytase challenged with glyphosate** showing few vacuolation and apoptotic bodies (thick arrow) beside minimal submucosal edema (arrowhead). Image magnification = 100x

Histopathological changes in the gills, liver, and intestine for different groups are shown in Figs. 2, 3 and 4. Control gills showed normal histological appearance. Each gill shows numerous gill filaments. Each filament is lined with epidermal epithelium, and each lamella consisting of pillar cells surrounding blood capillaries (Fig. 2A). The glyphosate Challenged group revealed diffuse lamellar fusion with lamellar congestion and hemorrhage (Fig. 2B and C). MOLE (Group 2) showed normal histological appearance (Fig. 2D). MOLE challenged (Group 2x) showed focal lamellar thickening with interlamellar congestion (Fig. 2E). MOLE and phytase (Group 3) showed lamellar congestion with focal lamellar fusion (Fig. 2F). MOLE and phytase-challenged (group 3x) showed moderate to severe lamellar thickening with lamellar fusion beside lamellar lifting (Fig. 2G).

Histopathological changes in the liver samples, control liver showed normal histological appearance of hepatic parenchyma and hepato-pancreas including normal central vein, hepatic cords, blood sinusoids, and presence of lipid droplets within the cytoplasm of hepatocytes (Fig. 3A). The glyphosate challenged group showed diffusely swollen hepatocytes with multifocal coalescing hepatocyte necrosis and inflammatory aggregates (Fig. 3B). Higher magnification shows necrotic hepatocytes surrounded with inflammatory aggregates beside diffuse hepatic vacuolation and sinusoidal congestion (Fig. 3C). The glyphosate challenged group also exhibited diffuse extensive hepatic vacuolation and minimal sinusoidal congestion (Fig. 3D). MOLE (Group 2) showed moderate hepatic vacuolation with sinusoidal congestion (Fig. 3E). MOLE challenged (Group 2x) showed diffuse hepatic vacuolation (Fig. 3F). MOLE and phytase (Group 3) showing moderate hepatic vacuolation (Fig. 3G). MOLE and phytase challenged (Group 3x) showed diffuse severe hepatic vacuolation (Fig. 3H).

The intestine of the control group of Nile tilapia fed the basal diet exhibited the normal histological appearance of intestinal villi (Fig. 4A). The glyphosate challenged group showed extensive apical desquamation with mild lamina propria inflammatory aggregates (Fig. 4B and C). The glyphosate challenged group showed diffuse shortened, stunted fused villi with marked lamina propria with cellular infiltrate and submucosal edema (Fig. 4D). MOLE (Group 2) showing normal histological appearance with increased villi length (Fig. 4E). MOLE challenged (Group 2x) showing mild intestinal vacuolation (Fig. 4F). MOLE and phytase (Group 3) show intestinal vacuolation with few invaded inflammatory cells (Fig. 4G). MOLE and phytase challenged (Group 3x) showed few vacuolation and apoptotic bodies besides minimal submucosal edema (Fig. 4H).

### Differential gene expression analysis

**Fig. 5:**
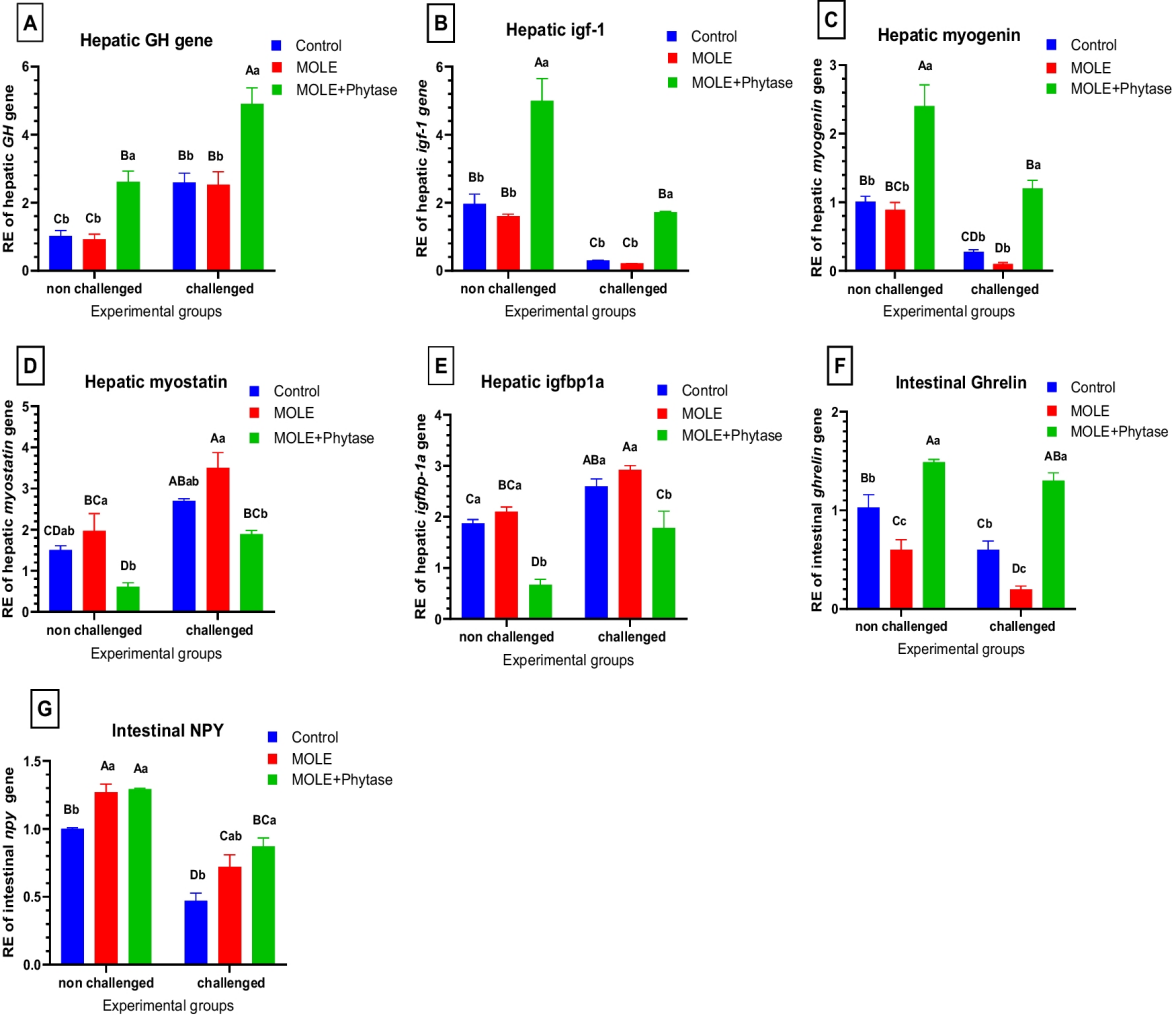
Relative gene expression of hepatic growth-related genes. GH: growth hormone gene, igf: insulin-like growth factor, igfbp: insulin-like growth factor binding protein, NPY: Neuropeptide Y. Columns with different superscript letters in the same figure are significantly different (*p* ≤ 0.05)

**Fig. 6:**
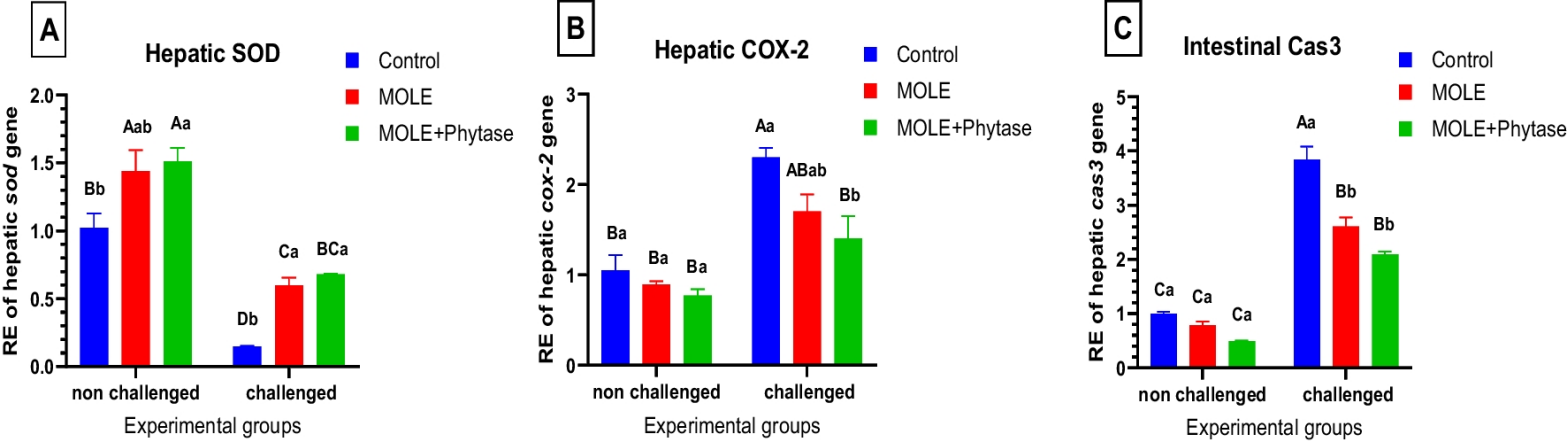
Relative gene expression of antioxidant-related (*sod*), and inflammatory-related genes (*cox2*) and apoptotic gene (*caspase-3*). Columns with different superscript letters in the same figure are significantly different (*p* ≤ 0.05)

The effect of dietary MOLE and phytase on the transcriptional levels of growth-related (*gh,igf1,igf-bp, myst, myogenin,gherlin and NPY*), antioxidant-related (*sod*), and inflammatory-related genes (*cox2*) and apoptotic gene (*caspase-3*) of normal Nile tilapia and three days after the glyphosate challenges are shown in Figs. 5 and 6. Generally, all studied genes showed a significant difference among the experimental groups.

#### Growth performance-related gene expression

Concerning the expression levels of liver growth-related genes including (*gh,igf1, myogenin, myst* and *igf-bp*) and intestinal *gherlin and NPY*; dietary supplementation of MOLE and phytase enhanced the transcriptional levels of liver *gh* (Fig. 5A), *igf1* (Fig. 5B) and *myogenin* (Fig. 5C), *gherlin* (Fig. 5F) and intestinal *NPY* (Fig. 5G) both pre and post glyphosate challenge compared with the control groups. However, dietary supplementation of MOLE and phytase markedly reduced hepatic *igf-bp* (Fig. 5E) and *myst* (Fig. 5D) in both pre and post-glyphosate challenge compared with the control groups. Meanwhile, fish group supplemented with MOLE only revealed a non-significant change in the hepatic expression level of *gh, ilgf*, myogenin, myostatin while exhibit a significant reduction in the intestinal gerlin with increased NPYa pre and post-challenge compared with the control groups.

#### Hepatic antioxidant genes expression

Concerning the expression level of liver antioxidant-related gene (*sod*); glyphosate challenged group revealed a marked decline in the expression level of hepatic *sod* compared with the control group. Whereas, dietary supplementation of MOLE either alone or with phytase enhanced the transcriptional levels of hepatic *sod* (Fig. 6A) in both pre and post-glyphosate challenge compared with the control groups.

#### Hepatic inflammatory response-related gene expression

Liver inflammatory-related genes including *cox2,* glyphosate challenged group exhibited a marked increase in the expression level of hepatic *cox2* paralleled with the control group. However, dietary supplementation of MOLE either alone or with phytase reduced the transcriptional levels of liver cox2 (Fig. 6B) in both pre and post-glyphosate challenge compared with the control groups.

#### Intestinal apoptosis-related gene expression

Liver apoptosis-related genes including *caspase 3;*glyphosate challenged group exhibited a marked increase in the expression level of hepatic *caspase3* compared with the control group. Moreover, dietary supplementation of MOLE either alone or with phytase downregulated the transcriptional levels of liver *caspase 3* (Fig. 6C) in both pre and post-glyphosate challenge compared with the glyphosate challenged group.

## Discussion

Water pollution with herbicide derivatives is a major worry that endangers the sustainability and value of ecosystems and aquatic animals (Blahova et al. 2020; Bojarski and Witeska 2020). Incorporated agriculture–aquaculture systems contribute directly to pollution with herbicides, which can adversely infleunce aquatic animals health status (Soror et al. 2021; Yousef et al. 2021). This research demonstrated that waterborne herbicides negatively impact the metabolic and biochemical functions of aquatic animals (Samanta et al. 2014; Abdel-Warith et al. 2021).

The plant extract utilized in our study was of the genus *Moringa*. The morphology of the leaves of *M. oleifera* and *M. stenopetala* are quite similar with few differences regarding the shape and apex of the leaves which could be variable according to the growth stage (Azza 2014; Boopathi and Abubakar 2021). In addition, wide variation in morpho-anatomical features of the *M. oleifera* accessions is reported. For this reason, we carried out a chemical investigation for assuring the species authentication. The plant was extracted with methanol (90%) to reduce the phytic acid, tannins and saponin glycoside content in the resulting extract as these compounds are soluble in water and less soluble in alcohol. The alkaloids detected in MOLE are determinant for *M. oleifera.* Saponin glycosides and tannins were not detected in MOLE by methanol solvent. Inositol monophosphate is a phytate compound and was detected in our study.

The growth parameters measured in our study (FBW, WG%, SGR, PER, and FCR) were considerably enhanced with the dietary inclusion of MOLE and phytase indicating significantly higher growth indices compared to other experimental groups. The performance improvement observed in the MOLE and phytase group probably attributed to the high content of bioactive compounds in MOLE, as p- quercetin, and kaempferol. These compounds are known to enhance the growth indices of numerous fish species, as common carp (*Cyprinus carpio*) (Ahmadifar et al. 2021), snakehead fish (*Channa argus*) (Kong et al. 2022), and grass carp (*Ctenopharyngodon idellus*) (Xu et al. 2023). The growth-promoting properties and health benefits of MO are linked to its rich array of bioactive components, including vitamins, flavonoids, phenolic acids, tannins, isothiocyanates, and saponins, that are abundant in various parts of the plant, particularly the leaves, and exert significant growth-stimulating effects (Vergara-Jimenez et al. 2017). Additionally, MOL contains high concentrations of antioxidants as vitamin C, β-carotene, and quercetin, that enhance the health situations of fish (Makkar and Becker 1997; Vergara-Jimenez et al. 2017).

Moreover, adding phytase to the MOLE-supplemented diet improves growth performance matched to control group. Debnath et al. (2005) observed that the inclusion of the phytase enzyme in Atlantic salmon diet improved its digestibility coefficients at an optimal dose of 500 FTU/kg. Hussain et al. (2014) noted that phytase can break down antinutrients in the diet, as phytic acid and trypsin inhibitors, thereby increasing nutrient digestion. Phytase enzyme converts such anti-nutritional substances into simple, definitely absorbed minerals (Sokrab et al. 2012). Similar conclusions were reached by Shahzad et al. (2017) and Hussain et al. (2017). When *C. catla* fingerlings were fed a diet with 900 FTU/kg of phytase, they recorded the utmost digestibility of crude protein (CP) and gross energy (GE). Similarly, Maas et al. (2019) perceived an enhancement of growth performance of Nile tilapia (*Oreochromis niloticus*) on a sunflower meal-based diet with 1000 FTU/kg of phytase.

Importantly, MOLE alone demonstrated a substantial decrease in growth and ration consumption when compared to control group. This reduction may be due to the antinutritional components as phytic acid (Spinelli et al. 1983; Richardson et al. 1985; Hossain and Jauncey 1993). These substances have been shown to reduce or hinder fish development performance. Moreover, phytate can affect the bioavailability of different minerals and decrease protein digestibility by forming complexex of phytic acid and protein, which can significantly harm the pyloric caecum by inhibiting nutrient absorption (Francis et al. 2001). previous researches have shown that 5–6 g of phytic acid per kg of diet can negatively affect the growth rate of common carp (Hossain and Jauncey 1993) and rainbow trout (Spinelli et al. 1983). Regarding total phenolics, a comparatively high levels of total phenolic compounds from mucuna beans in the diet of common carp has been recorded to considerably retard growth performance and feed utilization (Siddhuraju and Becker 2001). High quantities of total phenolic compounds have been shown to impair digestibility of protein and availability of amino acids by forming phenolic-protein and/or phenolic-protein-enzyme complexes. Furthermore, Hilton et al. (1983) also mentioned a similar inhibition in the growth performance of rainbow trout supplemented with a high-fiber diet. Similar studies also indicated that natural extracts like garlic oil supplementation had positive effects on growth, haematology, blood biochemistry, hepatosomatic index and histopathological parameters in Nile tilapia (Oreochromis niloticus) exposed to cypermethrin toxicity (Öz et al. 2024).

To understand the underlying mechanism influencing growth performance we evaluated the mRNA expression levels of some growth-related genes. In the current study, we found that MOLE supplemented group exhibited reduced FBW, BWG%, SGR with non-significant change of hepatic *gh, ilgf,* myogenin, *ilgfbps and* myostatin with raised NPYa pre and post-glyphosate challenge compared with the control groups. Interestingly, an opposite finding by phytase addition to MOLE was detected pre and post-glyphosate challenge compared with the control groups. Both GH (growth hormone) and IGF (insulin-like growth factor) are potent growth regulators that promote anabolic effects on carbohydrate and protein metabolism, intervening the actions of growth hormone (Perez-Sanchez and Le Bail 1999; Amin et al. 2019). An increase in ghrelin ultimately leads to increased availability of GH (Kojima and Kangawa 2005; Ceranowicz et al. 2010; Lewitt 2013). Tests performed in sheep by Sugino et al. (2002) showed that the infusion of amino acids and proteins enhances plasma ghrelin levels. According to Olaofe et al. (2013), *M. oleifera* contains great concentrations of essential amino acids as lysine, methionine, isoleucine, leucine, tryptophan, phenylalanine, threonine, and valine. MOLM has a high content of protein, ranging from 25% (Makkar and Becker 1996) to 32% (Soliva et al. 2005). *Moringa Oleirfera* is also rich in protein (32–35%), amino acids and essential vitamins (Hassan et al. 2018).

In teleost fish, growth and development of skeletal muscle are largely dependent on the availability of nutrient, that modulates the GH/IGF1 axis (Reinecke et al. 2005; Liu et al. 2020). Our study found that the expression levels of *gh, ilgf,* and myogenin were statistically non-significant in the fish group fed MOLE alone, both pre- and post-challenge. However, these levels were significantly elevated in the fish group fed both MOLE and phytase, pre- and post-glyphosate challenge. In contrast, *ilgf1bp1a* and myostatin levels were markedly raised in the glyphosate-challenged group, indicating that *ilgf1bp1a* and myostatin exert an inhibitory effect on *gh, ilgf*, and myogenin activities in Nile tilapia challenged with glyphosate.

Herein, the increase in growth and diet utilization observed in this study by phytase addition to MOLE may be attributed to the immune-nutritional components of *M. oleifera* and its ability to enhance feed digestibility, absorption, and assimilation. This is achieved through the augmented digestive enzymes and healthy intestinal microflora promoted by *M. oleifera*'s prebiotic activity (Nkukwana et al. 2014).

Biochemical indices serve as valuable biomarkers for evaluating both physiological and health conditions of fish, evaluating the nutritional value of fish diets, and evaluating the impacts of hazardous compounds (Aliko et al. 2018; Khafaga et al. 2020). Meanwhile, liver injury biomarkers such as ALT and AST enzymes and kidney indices including blood urea nitrogen and creatinine levels were significantly elevated in the glyphosate-intoxicated group. These results indicate the occurence of hepatotoxicity, liver dysfunction, and hepato-renal damage in fish exposed to glyphosate (Bacchetta et al. 2014). The results are in line with Yousef et al. (2021), who detected high serum activities of AST and ALT in common carp (*Cyprinus carpio*) subjected to glyphosate. Increased urea levels are associated with gill damage, while high creatinine levels are linked to muscular dysfunction (Soror et al. 2021). The declined concentration of total proteins, albumin, and globulins due to glyphosate toxicity, indicates liver tissue damage from oxidative stress (Brum et al. 2018).

TC, TG, and VLDL-C levels were elevated in the glyphosate-intoxicated group of Nile tilapia. Dietary inclusion of MOLE modulated lipid profiles in tilapia by prompting a hypolipidemic effect. Monir et al. (2020) and El-Kassas et al. (2020a, b) revealed a substantial reduction in serum triglyceride (TG) and total cholesterol (TC) values in Nile tilapia-fed MOLE-based diets, other researchers indicated that allspice supplementation at 10 g kg^−1^ for 60 days, has adequate beneficial effects by improving the haemato-immunological and biochemical status of O. mossambicus after acidic stress (Yılmaz et al. 2015).

The reduced serum TC levels may be accredited to the presence of β-sitosterol (Vergara-Jimenez et al. 2017), that inturn decreases the absorption of endogenous cholesterol, enhances its secretion from the gastrointestinal tract, and facilitates its excretion as neutral steroids (Mehta et al. 2003). Furthermore, the lowered levels of serum cholesterol, triglycerides, and lipoprotein could be accredited to the suppression of cholesterol synthesis, leading to a diminution of liver intracellular sterols (Mehta et al. 2003).

Oxidative stress occurs in case of overproduction of ROS and the body’s endogenous antioxidant mechanisms are unable to effectively scavenge and neutralize these ROS (Nordberg and Arnér 2001; Le Bras et al. 2005).

Long-term exposure to glyphosate-based herbicides induce excessive production of ROS and oxidative stress in fish species (Li et al. 2017). This exposure inhibits the activities of catalase (CAT), superoxide dismutase (SOD), and level of glutathione (GSH) in the gills of common carp and the liver of *Prochilodus lineatus* (Modesto and Martinez 2010; Ma et al. 2019). Similarly, reduction was detected in the serum, gills and liver of Nile tilapia exposed to higher glyphosate concentrations (4 and 16 mg/L). Acute glyphosate exposure also led to lipid peroxidation in the neotropical fish *Prochilodus lineatus* (Modesto and Martinez 2010) other studies also indicated that antioxidant enzyme-related genes (SOD, CAT, GPx, and GST) were significantly up-regulated to suppress oxidative stress while the expression levels of immune-related genes (TGF-β, TGF-α and IL1-β) decreased in Nile tilapia exposed to a dose of ≥ 20 mg/L GBH (Acar et al. 2021).

Cellular antioxidants are crucial components of tissues that protect cells against oxidative damage induced by ROS (Hamed et al. 2022). SOD transforms superoxide radicals into dioxygen and hydrogen peroxide, which catalase additional breaks down into water and molecular oxygen (Akhigbe and Ajayi 2020). Sreelatha and Padma (2009), Charoensin (2014) demonstrated that MOLE exhibits a robust scavenging influence on 2,2-diphenyl-1-picrylhydrazyl (DPPH) free radicals, superoxide radicals, and nitric oxide radicals, thereby protecting biomolecules from oxidative damage.

However, MOLE, either alone or with phytase, was able to enhance hepatic *sod* gene expression both before and after glyphosate challenge compared with the control groups. These antioxidant properties of MOLE might be due to its content of considerable level of phytogenic components, which protect against oxidative stress-associated damage (Kou et al. 2018).

In this regard, the high content of ascorbic acid, phenolics, and flavonoids such as quercetin, and kaempferol in Moringa leaf extract helps restore levels of glutathione, glutathione peroxidase (GPx), glutathione-S transferase (GST), and glutathione reductase (GR) by preventing lipid peroxidation and scavenging free radicals (Sharifudin et al. 2013; Singh et al. 2014; Xu et al. 2019).

Oxidative stress is identified as both a cause and a result of inflammation. It prompts the translocation of NF-kB into the nucleus, leading to the transcription of various harmful pro-inflammatory genes (Won et al. 2006; Zheng et al. 2017; Hamed et al. 2022). Additionally, oxidative stress enhances pro-inflammatory cytokines including *tnf-α* and *il-6*, which then activate *nf-kb* (Akhigbe and Ajayi 2020; Hamed et al. 2022). Ma et al. (2019) revealed that glyphosate treatment increased NF-κB levels in the gill tissue of common carp, likely due to the accumulation of excess ROS. These results are consistent with earlier studies in which the *tnf-α* and *il-1β* genes were upregulated in the gills of carp following exposure to glyphosate (Ma et al. 2019; Wang et al. 2020). In the same time, Tang et al. (2020) recorded an increase in *il-1β, il-6* and *tnf-α* in different parts of rat small intestine after Glyphosate exposure. These findings concluded that glyphosate application triggered the NF-κB pathway, resulting in increased secretion of pro-inflammatory cytokines, that initiated an inflammatory response. The current study revealed that glyphosate-challenged group showed a marked increase in hepatic *cox-2* expression. However, the inclusion of moringa and phytase successfully reduced its expression level. Fard et al. (2015) reported that M. oleifera hydroethanolic bioactive leaf extracts significantly reduced *cox-2* protein expression and suppressed the *nf-κb* signaling pathway, proposing the potent activity of *M. oleifera* bioactive leaf extract as an inhibitor of inflammatory cytokines and mediators.This finding aligns with the fingings by Xu et al. (2019), which revealed the anti-inflammatory and antioxidant effects of crude extracts from *M. oleifera* leaves.

Hence, the antioxidative and anti-inflammatory properties of MOLE observed in this study can be ascribed to its phenolic compounds and flavonoids which potentiate MOLE to be beneficial in preventing glyphosate-induced toxicity.

ROS up-regulate genes that encode redox-regulated transcription factors that are correlated with the initiation stage of apoptosis (Sinha et al. 2013). The caspase-cascade system plays an important role in the induction, transduction,and amplification of intracellular apoptotic signals. At the beginning of apoptosis, initiator caspases (caspase-8 and −9) cleave and activate downstream effector caspases (caspase-3, −6, and −7), which directly cause apoptosis. In this study, hepatic expression levels of caspase 9 and 3 were upregulated implying that GLY can induce apoptosis in the liver of fish. In the intrinsic pathway, the critical point in the apoptotic signaling progression is the release of cytochrome c from mitochondria after the collapse of mitochondrial membrane potential (Jaeschke et al. 2018). Once cytochrome c binds with apoptotic protease activating factor 1 (Apaf-1) and ATP, leading to the initiation of the caspase cascade reactions that activates caspase-9 and caspase-3 which can destroy the cells. Hao et al. (2019) stated that the concentration of GLY increases; the discharge of cytochrome c into the cytoplasm increases.

Intoxicated fish by glyphosate upregulated intestinal caspase-3 expression which was ameliorated by MOLE administration through its antioxidant and anti-inflammatory properties confirming the potential antiapoptotic impact of MOLE. The phenolic compounds in MO Leaves act as anti-inflammatory, antioxidant, and antiapoptotic factors (Shahidi and Yeo 2018). Pérez-Galán et al. (2006) concluded that the excess ROS can prompt apoptosis in cells. MO leaves, rich in antioxidants and cytoprotective natural compounds, demonstarte a promising future strategy for reducing abnormalities associated with cellular peroxidative damage and apoptosis (Lin et al. 2021; Khalid et al. 2022). Ma et al. (2019) indicated that, the expression level of bax is increased, while bcl-2 is decreased in the gills of glyphosate-treated fish. This indicates that activating *bax* and inhibiting *bcl-2* could initiate the caspase cascade reaction via the mitochondria-mediated pathway in the gills of carp following exposure to glyphosate (Miest et al. 2012). In this study, a significant elevation in mRNA level of caspase3 was recorded in the glyphosate-treated group, this indicates that glyphosate exposure initiated mitochondria-mediated apoptosis in fish intestine, which is also revealed by Sulukan et al. (2017) who demonstrated that glyphosate exposure caused cellular death in zebrafish. *M. oleifera* leaf ethanolic extract (MLEE) decreased the expression of *caspase-3* and *bax* genes while increasing the expression of *bcl-2* genes, hence improving mitochondrial membrane potential (Samie et al. 2018; Abdel-Daim et al. 2020) owing to MLEE’s anti-apoptotic properties. Meanwhile, reproductive deficits induced by alcohol in male rats were attenuated by phenolic substances such as p-coumaric acid, which reduced the immunoreactivity of *caspase-3*, *caspase-7*, and *p21* (Nishi et al. 2018).

Histology studies are considered a sensitive method for diagnosis of organ toxicity due to xenobiotics (Lanning et al. 2002). They offer detailed information on the acute and chronic impacts of toxicants on specific organs (Amacher et al. 2006). The histological changes reported in our analysis are consistent with earlier investigations, demonstrating that glyphosate exposure promotes unfavorable cytological modifications in the gills, liver, and intestine.

Fish gills are the principal location for ion exchange with the environment, as well as the major channel of pesticide penetration, because they are constantly in contact with water (Abdel-Moneim et al. 2012; Tabassum et al. 2016; Soare et al. 2019).

In the current experiment, the glyphosate-challenged group exhibited diffuse lamellar fusion, lamellar congestion, and hemorrhage. This is in accordance with the findingd by Ma et al. (2019) who concluded that exposure to 104.15 mg L^−1 of glyphosate for 7 days resulted in significant damage to fish gills. Similar observations have been observed in neotropical fish (Shiogiri et al. 2012), Nile tilapia (Jiraungkoorskul et al. 2002; Samanta et al. 2017), *Poecilia reticulata* (Rocha et al. 2015), and Bloch (Samanta et al. 2016), revealing that exposure to glyphosate leads to gill damage in Nile tilapia, ranging from mild to severe lamellae necrosis, congestion, and hemorrhage.

In this study, the glyphosate challenged group showed diffusely swollen hepatocytes with multifocal coalescing hepatocyte necrosis and inflammatory aggregates. In line with, Mohapatra et al. (2020); Verma et al. (2020) who stated that the most frequent negative consequences of pesticide exposure on the liver include necrosis, cellular deformity linked to nuclear hypertrophy and vacuolization, sinusoidal enlargement, and congestion of sinusoidal spaces caused by WBC infiltration. Similarly, Barbhuiya and Dey (2014) who observed that *Heteropneustes fossilis* had central venous congestion, hepatocyte degradation, cytoplasmic vacuolization, vacuoles in sinusoids, and hepatocytes with pyknotic nuclei after 21 days. On exposure to 5, 10 and 20 ppm concentrations of an organophosphate pesticide for 25 days, *Heteropneustes fossilis* showed cytoplasmic degeneration, pyknosis in liver tissues, vacuoles in hepatic cells, and rupture in hepatic blood vessels (Islam et al. 2019).

Furthermore, glyphosate challenged group showed extensive apical desquamation with mild lamina propria, inflammatory aggregates with diffuse shortened, stunted fused villi with marked lamina propria cellular infiltrate, and submucosal edema. Begum et al. (2013) reported that *Heteropneustes fossilis* subjected to 7 ppm of arsenic concentration for 15 days had degenerated villi, and at 20 ppm, damaged serosa was detected, leading to mucosal fusion and edema. Further Islam et al. (2019) reported that *Heteropneustes fossilis* when subjected to organophosphate pesticide, showed swelling, disintegrating sub-mucosa, mildly damaged serosa, and fused or ruptured villi at concentrations ranging from 20 to 25 ppm. Interstingly, Shahzadi et al. (2024) detected ameliorative effects of *Moringa oleifera* against carbofuran induced toxicity in rohu *Labeo rohita*.

## Conclusion

In summary, the findings indicate that MOLE and phytase significantly improved growth performance and feed utilization while offering protection against glyphosate-induced kidney and liver toxicity and oxidative stress. This protective action is linked to their ability to modulate antioxidant, anti-inflammatory, and anti-apoptotic signaling pathways, owing to their rich content of bioactive compounds such as polyphenols, flavonoids, and essential nutrients.

## Data Availability

No datasets were generated or analysed during the current study.
